# Effects of Sexual Dimorphism and Landscape Composition on the Trophic Behavior of Greater Prairie-Chicken

**DOI:** 10.1371/journal.pone.0079986

**Published:** 2013-11-11

**Authors:** Beatriz Blanco-Fontao, Brett K. Sandercock, José Ramón Obeso, Lance B. McNew, Mario Quevedo

**Affiliations:** Research Unit of Biodiversity, (UO/CSIC/PA), Asturias, Spain; Dpt. Biología de Organismos y Sistemas, Área de Ecología, Universidad de Oviedo, Asturias, Spain; Division of Biology, Kansas State University, Manhattan, Kansas, United States of America; University of Lethbridge, Canada

## Abstract

Partitioning of ecological niche is expected in lekking species that show marked sexual size dimorphism as a consequence of sex-specific ecological constraints. However, niche partitioning is uncertain in species with moderate sexual dimorphism. In addition, the ecological niche of a species may also be affected by landscape composition; particularly, agricultural fragmentation may greatly influence the trophic behavior of herbivores. We studied trophic niche variation in Greater Prairie-Chickens (*Tympanuchus cupido*), a grouse species that shows moderate sex-dimorphism. Greater Prairie-Chickens are native to tallgrass prairies of North America, although populations persist in less natural mosaics of cropland and native habitats. We used stable isotope analysis of carbon and nitrogen in blood, claws and feathers to assess seasonal differences in trophic niche breadth and individual specialization between male and female Greater Prairie-Chickens, and between birds living in continuous and fragmented landscapes. We found that females showed broader niches and higher individual specialization than males, especially in winter and autumn. However, differences between females and males were smaller in spring when birds converge at leks, suggesting that females and males may exhibit similar feeding behaviors during the lekking period. In addition, we found that birds living in native prairies showed greater annual trophic variability than conspecifics in agricultural mosaic landscapes. Native habitats may provide greater dietary diversity, resulting in greater diversity of feeding strategies.

## Introduction

Size dimorphism in vertebrates is usually associated with polygamy and differences between the sexes in reproductive role [1]. In lekking species (*i.e.*, those whose males compete at displaying grounds known as “leks” for the favor of females), larger body size in males may be the result of sexual selection [2], [3].

Niche partitioning often occurs in species that show marked differences in size between males and females and each sex may have distinct nutritional requirements [4]–[8], resulting in differences in niche breadth and individual diet specialization [7], [9], [10]. However, size dimorphism decreases from large to small-bodied lekking species [11]. Thus, niches of lekking species without differences in size between females and males should overlap. Alternately, females and males may partition their niche as a result of distinct reproductive roles in their polygynous mating system [12], [13].

The niche of a species is also expected to vary throughout its distribution [14], partly due to variation in landscape composition [15], [16]. Fragmentation of native habitats due to conversion to agricultural crops is one of the main causes of landscape change worldwide, often resulting in mosaics of agricultural lands and native habitat [17], [18]. Such landscape changes influence ecosystem dynamics by shifting species' trophic niches due to changes in the availability of resources [19]–[22].

In grouse (Tetraonidae), sexual dimorphism in body size ranges from large (100%) in Capercaillie (*Tetrao urogallus*) to small (17%) in prairie grouse [23], [24]. Previous work has shown different aspects of niche segregation in Capercaillie [4], [25]. In contrast, Greater Prairie-Chicken (*Tympanuchus cupido*; hereafter *prairie-chicken*) males and females are expected to show less niche partitioning than Capercaillie based on the smaller size differences between sexes (e.g. [26])- similar to that found in most monogamous birds (10–15%; [13]). Nevertheless, reproductive roles in lekking species are sex-specific and males do not provide parental care or associate with females outside of the breeding season [27]. This reproductive behavior may prevail over the degree of size dimorphism to drive niche segregation.

Prairie-chickens are native to prairies of central North America and were widespread prior to European settlement [24]. However, cropland expansion and large-scale agricultural transformations largely fragmented their native habitat [28]. Today the core range of prairie-chickens extends from South Dakota to Oklahoma [24], including both areas dominated by native grasslands and others where remnants of tallgrass prairies are embedded in a cropland matrix. Previous studies suggest that variability in landscape composition and human land use has resulted in large differences in demography, genetic structure, and population viability of prairie-chickens in the core of the range [29]–[31]. Hence, niche differences between environments (i.e. native grasslands *vs* remnants of tallgrass prairies embedded in a cropland matrix) might be expected due to differences in habitat structure and resource quality and availability.

Trophic ecology is a central aspect of a species' ecological niche. The ability of animals to forage is a large part of their ability to cope with the environment, thus influencing behavior, habitat selection, and demography [32]. Populations may respond to biotic and abiotic conditions of their local environment with shifts of their trophic behaviors [33], [34]. Different aspects of variation in trophic behavior among populations may be determined by differences in average diet composition, and diet variance [35]–[37]. For instance, an important source of variation of a species' trophic ecology is how much individuals vary their food resources across the species range [35], [38]. Intrapopulation variation can be attributed to sex or age-class differences, or to individual specialization [10]. Prairie-chickens are at the lower end of the size range of endothermic herbivores and staple foods such as native grass and forbs are often abundant but of low-quality [39]. Therefore, individuals have to devote extra time to feeding compared to larger herbivores, such as ungulates, to meet their energy requirements [40], [41].

Stable isotope analysis of consumer tissues is a powerful tool to study intrapopulation niche partitioning [42], [43]. The stable-isotope ratio of a given tissue reflects the diet during its synthesis [44], [45]. Hence, analyzing tissues with different growth and turnover rates provides diet information that spans several temporal scales. We used stable isotope analyses of feathers, claws and whole blood to gain insight into autumn, winter and spring diets, respectively. Specifically, we aimed to (1) evaluate intra-specific dietary variation between females and males throughout the annual cycle, and (2) compare prairie-chicken feeding behavior in two populations differing in the amount of native habitat in their range. We show sexual niche segregation in a lekking species with moderate sexual size dimorphism and niche differences between birds living in native prairies and birds living in agricultural mosaic landscapes.

## Methods

### Study sites

**Figure 1:**
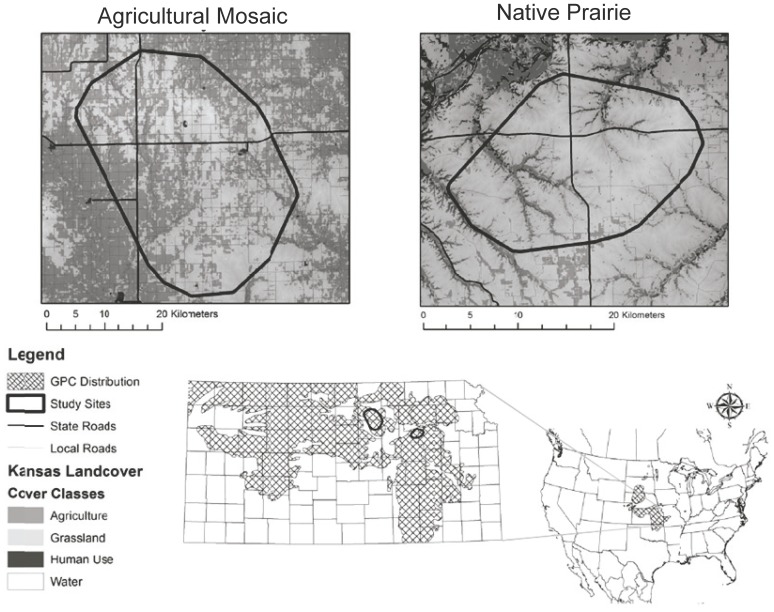
Greater Prairie-Chicken distribution and land cover of the study sites in Kansas showing differences in grassland fragmentation. The Smoky Hills study site is a mosaic of agricultural fields and native prairie. Native tallgrass prairie is relatively unfragmented at the Flint Hills site.

Our study was conducted at two sites that differed in land cover, land use and degree of fragmentation of native tallgrass prairie habitat. The two sites were located 110 km apart in the northern Flint Hills and the Smoky Hills of north-central Kansas, U.S.A. (Fig. 1). The Flint Hills area (hereafter *native prairie*; 533 km^2^; 39°00′N 96°26′W) was covered by 81% grassland and 10% cropland, with an average patch size of grassland of 51 ha, and a road density of 0.57 km per km^2^. Grasslands at the Smoky Hills site (hereafter *agricultural mosaic*; 1,295 km^2^; 39°25′N 97°34”W) were more fragmented, with 53% cover of grassland and 38% cropland, smaller average size of grassland patches (15 ha), and higher road density (1.4 km per km^2^). Crops within the agricultural mosaic included sorghum, wheat, corn and soybean. The density of prairie-chickens was similar in both sites: 0.19 and 0.17 birds km^−2^ in the native prairie and the agricultural mosaic, respectively [30], [31].

### Tissue collection

Prairie-chickens were captured at 20 leks between March and May 2009, by means of walk-in traps and drop-nets [46], [47]. At first capture, we collected samples of three tissues: blood, claw and feather samples. We clipped 3 mm of the distal part of a claw and stored it in a sterile envelope. Cutting the tip of the claw allowed us to collect 1 ml of blood from the toe vein in an Eppendorf tube, preserved in 70% ethanol. Last, a covert feather from the breast was cut and stored in a paper envelope.

**Figure 2:**
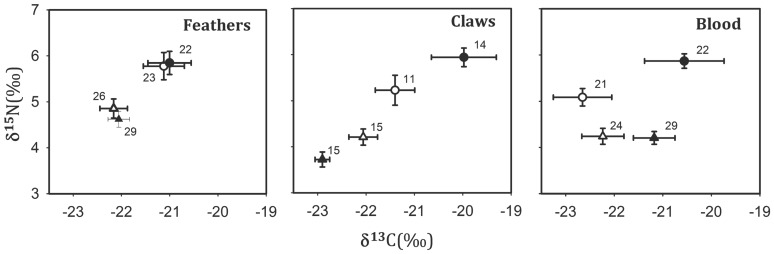
Stable isotopes of carbon and nitrogen (δ13C; δ15N; mean ± SD) in feathers, claws and blood of Greater Prairie-Chickens in Kansas, 2009. Filled symbols  =  males, open symbols  =  females. Triangles  =  agricultural mosaic, circles  =  native prairie. Numbers in parentheses indicate sample size.

We captured 156 prairie-chickens at 8 leks located in native prairie and 12 leks located in agricultural mosaic between 6 March and 6 May, 2009. All samples from birds captured in the native prairie (n = 48) were used in isotopic analysis, whereas from birds captured in the agricultural mosaic (n = 114) we used a 50% random selection of the males and all the females (n = 57), to balance sample sizes. The selection totaled 96 blood samples, 55 claw samples, and 100 feather samples distributed across sex-classes and study sites (Fig. 2).

In addition we collected vegetation samples to establish the isotopic baseline for prairie-chicken (see methods and analysis details in Text S1; Tables S1 and S2; Fig. S1).

### Stable isotope analysis

We measured *δ* ^13^C and *δ* ^15^N ratios in blood, claws and feathers to examine sex- and habitat-related partitioning of trophic niche. Each tissue integrates dietary information from different periods of the annual cycle: whole blood has rapid turnover, and integrates the isotopic values of the diet during the 3–6 wk prior to sampling [48]. Hence, whole blood integrates prairie-chicken *spring* diet. Claws are a metabolically inert tissue that grow continuously and integrates diet over longer periods. The tip of claws integrates dietary information of a 2–5 month period before sample collection, which corresponds to *winter* diet [49]. Last, feathers are metabolically inert tissues that preserve diet information at the time of their formation, which in this case corresponds to the *autumn* diet of the previous year during molt [44], [45], [49].

Blood samples were dried to constant mass in an oven at 60°C, and then refrigerated until isotopic analysis. Feathers and claws were treated with a 2∶1 chloroform-methanol solution for 24 hours to remove oils and debris, and then dried in an oven at 60°C. Feathers were frozen in liquid nitrogen and immediately ground to fine powder using a MM200 ball mill. Approximately 1 mg (1±0.2 mg) of each of the tissues was subsampled and packed into a 4×6 mm tin capsules for *δ* ^13^C and *δ* ^15^N analyses using a continuous-flow isotope ratio mass spectrometer at the University of California Davis Stable Isotope Facility (USA). Stable isotope ratios are expressed in δ notation, as parts per thousand deviations from a standard (‰). We used Pee Dee belemnite limestone as a standard for δ^13^C and atmospheric nitrogen as a standard for δ^15^N according to the equation: *δX*  =  [(R_sample_/R_standard_) – 1]×1000 where *X* is ^15^N or ^13^C and R is the ratio of stable isotopes (^15^N:^14^N or ^13^C:^12^C).

A random quarter of the samples were analyzed in duplicate, and the analytical error was minimal at 0.13‰ (±0.14) for *δ* ^15^N and 0.18‰ (±0.24) for *δ* ^13^ C.

### Trophic niche breadth and variability

To calculate trophic niche breadth and trophic variability we used quantitative metrics based on the position of individuals and Euclidean distances among them in the *δ* ^13^C-*δ* ^15^N space [50]. Previously, *δ* ^15^N and *δ* ^13^C were standardized (z-scores) to avoid differential weighting of the axes in the *δ* ^13^C-*δ* ^15^N biplot [51], [52]. We applied those metrics to individuals at our two study sites and to males and females within each location. To estimate the total niche of each subset we measured the total area of the convex hull (*TA*) that included the isotopic values of all individuals.

To estimate the trophic variability within each sex and population we calculated Euclidean distances among individuals in the *δ* ^13^C-*δ* ^15^N plane. First, we calculated the distance of each individual to the isotopic centroid of its subset (*CD*), providing an index of the trophic diversity within each segment of the population. Second, we calculated the coefficient of variation of the distances from each individual to its neighbors in the isotopic space (*CVND*), which yields a measure of the clustering of values and trophic redundancy within the subset [53]. Populations or subsets of the population with a large proportion of individuals characterized by similar trophic ecologies should exhibit a smaller *CVND* (increased trophic redundancy) than a population in which individuals are, on average, more divergent in terms of their trophic niche [50], [53]. *CD* and *CVND* were calculated with Quantum Gis 1.6.0.

### Statistical analyses

**Figure 3:**
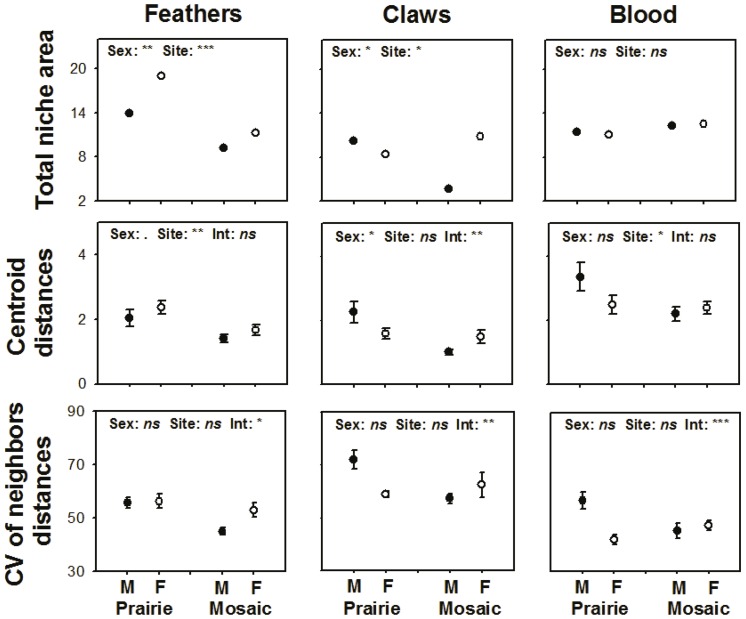
Trophic niche metrics (mean ± SE) and comparisons between Greater Prairie-Chicken populations occurring in native prairies (Prairie) and in an agricultural mosaic (Mosaic) and between males (M; black dots) and females (F; white dots). Total niche area (TA) was the area of the convex hulls that included δ13C-δ15N isotopic values of each of the subsets; Centroid distance (CD) was the mean distance of each individual to the isotopic centroid of its subpopulation; and the CV of the neighbors distances (CVND) was the coefficient of variation of distances from each individual to its neighbors in the isotopic space. P values of the differences between males and females and populations in native prairie and agricultural mosaic habitats in TA were estimated as the proportion of resampled data sets that exceeded the observed difference. Significance codes: *** p<0.001; ** p<0.01; * p<0.05;. p<0.1; ns = p>0.1

We fitted GLM models in R [54] to compare both absolute isotopic values and standardized metrics (*CD* and *CVND*) between males and females, and also between populations in different landscapes (Table 1; 2 Fig. 3). For each response variable, we fitted first the full model, i.e., including sex, landscape (agricultural mosaic or native prairie study sites) and the interaction, and proceeded to obtain a minimal model by removing non-significant interactions, then explanatory variables. We used gaussian errors in models fitted to absolute isotopic values, and gamma errors in models fitted to CD and CVND. We chose between the two error distributions by inspecting both density distribution of the data and Q-Q plots (e.g., [55]), the latter to evaluate the assumption of residuals approximately following a normal distribution. Where appropriate, we switched to the gamma distribution because it can be used for a continuous, positive response variable, and it describes right-skewed data distributions better than the normal [55], [56].

**Table 1:** Stable isotopes of carbon and nitrogen (*δ* ^13^C; *δ* ^15^N; mean ± SD) in feathers, claws and blood of Greater Prairie-Chickens in Kansas, 2009.

		Feathers	Claws	Blood
Study site	Sex	*δ* ^13^C	*δ* ^15^N	*δ* ^13^C	*δ* ^15^N	*δ* ^13^C	*δ* ^15^N
Agricultural mosaic	Male	−22.05±1.20	4.62±0.94	−22.88±0.57	3.74±0.90	−21.17±2.30	4.21±0.75
	Female	−22.16±1.46	4.86±1.06	−21.77±1.47	4.25±0.92	−22.23±2.12	4.24±0.85
Native prairie	Male	−21.00±2.10	5.85±1.17	−19.98±2.51	5.94±0.75	−20.50±3.80	5.87±0.73
	Female	−21.05±2.04	5.78±1.41	−21.40±1.36	5.23±1.08	−22.65±2.12	5.09±0.86

Total niche area (TA) itself is a summary variable, which yields a single value for each season (tissue) and landscape. To test for differences in *TA*, we randomized 1000 times the empirical data set of isotopic signatures and calculated *TA* in each resample to obtain a null distribution of *TA*. *P-values* were then calculated as the proportion of resampled data sets that exceeded the observed differences [57].

### Ethics statement

Sampling methods were approved by Kansas State University's Institutional Animal Care and Use Committee (Protocol numbers 2474 and 2781). Research occurred exclusively on private lands with landowner permission. Field study did not involve endangered or protected species.

## Results

### Differences between males and females

We found significantly greater *δ* ^13^C values in blood (spring) for males than for females, no differences in isotopic values of feathers (autumn) and a significant interaction for both isotopic values in claws (winter) and for *δ* ^15^N in blood (Table 1, 2; Fig. 2).

**Table 2:** Results of the GLM models (Gamma error distribution) comparing the effects of site, sex, and an interaction on the isotopic signatures of three tissues of Greater Prairie-Chickens (*Tympanuchus cupido*).

	Feathers	Claws	Blood
Variables	*δ* ^13^C	*δ* ^15^N	*δ* ^13^C	*δ* ^15^N	*δ* ^13^C	*δ* ^15^N
Study site	3.072**	4.72***	1.87**	2.71**	----	3.55***
Sex	----	----	----	----	2.68**	----
Interaction	----	----	2.86**	2.49*	----	2.50*
df	1, 98	1, 98	1, 51	1, 51	1, 94	1, 92

We report *t-values* for the most parsimonious (minimal) models *** p<0.001; **

p<0.01; * p<0.1. See figure 2 for sample size.

Nonparametric permutation tests showed that total niche space (*TA)* was wider in females, both in autumn (feathers; 23% wider) and in winter (claws; 27%). Total niche space was similar for both sexes at each site during the spring (Fig. 3). In autumn (feathers) and winter (claws), females showed greater distance of each individual value to the isotopic centroid (*CD*) than males. In winter the interaction between study site and sex for *CD* was significant. *CD* did not show differences between males and females in spring. *CVND* did not differ between males and females in any comparison (Fig. 3).

### Differences between landscapes

Total niche space (*TA)* was 38% broader in the native prairie than in the agricultural mosaic in autumn (feather samples) and 22% broader in winter (claw samples), whereas no differences were found for during the spring (blood; Fig. 3). The distance of each individual to the isotopic centroid (*CD*) was significantly higher for prairie-chickens in the native prairie than in the agricultural mosaic both in autumn (feathers) and spring (blood). *CD* in winter (claws) and the coefficient of variation of the distances from each individual to its neighbors in the isotopic space (*CVND*) did not differ between landscapes and showed a significant interaction between study site and sex in all comparisons of the three tissues (Fig. 3; glm; Gamma error).

## Discussion

Greater Prairie-Chickens showed sexual niche segregation during different periods of their annual cycle, despite only moderate differences in body size. We found intrapopulation partitioning of trophic niche between sexes, suggesting that females and males used partially different resources in autumn and winter, with females showing wider variability of resource use. In addition, we found that birds inhabiting the native prairie landscape showed wider trophic variability than birds living in the agricultural mosaic, although this should be interpreted with caution (see below).

Generally, wider niches were attained via an increase in individual diversity, supporting the niche variation hypothesis which states that more generalized populations are expect to be more heterogeneous [58], [59].

### Trophic sexual dimorphism

Our study provides the first evidence of sexual niche partitioning in North American prairie-grouse which resembles patterns found in old-world forest grouse [4]. We found that females and males showed distinct trophic variability in autumn that was diluted as spring approached (Fig. 3). Such seasonal differences suggest similar short term trophic behavior during the lekking season, when males and females converge at leks [24], [30], [60], and substantial dietary segregation among individuals from different sexes in the other seasons.

In autumn, wider niches and higher trophic diversity (Fig. 3) of females are consistent with a more generalized diet, based on greater individual variability of feeding strategies. Our inferences from stable isotopes agree with previously described aspects of the natural history of this species. Prairie-chickens tend to gather in flocks in autumn, in particular in feeding grounds, but females are more prone to solitary behavior [60]. Thus, female individuals might use distinct feeding grounds and dietary resources. In addition, females can be a more heterogeneous segment of the population in early autumn because females use habitats with good access to arthropods as a protein-rich diet for developing chicks, whereas non-breeders might select other habitats, for instance to minimize predation risk [60].

In winter, we found that differences between sexes were site-specific: females showed wider niches and higher trophic diversity in the agricultural mosaic but not in the native prairie landscape. Males had wider niches and higher trophic diversity in native prairie. We speculate that the reduced availability of diet resources in winter favors opportunistic behaviors. In the agricultural mosaic landscape, different kinds of cultivated grains become available at different times of the season [60]. Females, which show larger movements during winter, may be taking advantage of a greater variability of grains in the agricultural mosaic [60]. Conversely, males show shorter winter movements and remain closer to leks that occur predominantly in native grassland habitats [29], [60] and thus, greater niche width of males in native prairie may reflect a higher variety of native tallgrass seeds around leks [61].

Convergence of females and males on leks in the spring reduces sexual spatial segregation, likely resulting in similar diet resource use during the spring breeding season.

Among grouse, sexual differences in trophic strategies have been found for the most sexually dimorphic species of Capercaillie [4]. Although prairie-chickens have less sexual size dimorphism, males and females also show marked distinct reproductive roles [27], which may be the responsible of trophic niche divergence, following the sexual selection hypothesis [12], [13].

### Is prairie fragmentation a driver of feeding behavior?

We observed that prairie-chickens showed distinct foraging strategies in two landscapes that differed in the degree of native habitat loss and fragmentation. Generally, the population in the native prairie landscape showed more generalist foraging behavior and a higher individual trophic diversity than prairie-chickens occurring in an agricultural mosaic.

Landscape composition and the reduction of native habitat influence availability of food resources for herbivores. Hence, the higher trophic variability found in the native prairie landscape might indicate greater specific and microhabitat structural diversity of less fragmented native habitats [62], [63]. Conversely, individuals in the agricultural mosaic landscape may converge on the highly available resources provided by cultivated crops [64], resulting in generalist individual behavior and smaller population niches. Nevertheless, our lack of replicated study sites suggests a cautious interpretation of the landscape results, especially for the TA metric [65].

Populations adapt to local conditions and distinct trophic behaviors which depart from prevalent view of the species' niche, might be among such local adaptations [66]. Grouse species (Tetraonidae) often specialize in diet resources [67]. However, our results suggest that habitat conditions and breeding strategy may influence trophic niche dynamics, both within and among populations.

### Isotopic caveats

Stable isotope analyses do have several advantages over traditional techniques to study the population trophic structure of threatened or declining species (reviewed in [68], [69]. Layman et al. (2007) developed isotopic metrics to disentangle the isotopic structure of a population [50]. These metrics can offer a robust assessment of trophic structure in communities and populations once two assumptions are met: (1) *δ* ^15^N and *δ* ^13^C are appropriately scaled to have equal weighting when combined in metrics, (2) the variation in isotopic ratios of basal sources should be considered [51]. We solved the first of the limitations by standardizing the isotopic values (see Methods). The second one is a caveat that could affect our results related to the intrinsic isotopic variance in resources especially between the two study sites, and to different metabolic routing of distinct diet components [70]–[72]. In our study we assumed similar variability in both study sites (see Table S2) and minimized potential biases due to intrinsic resource variance by including study site as a variable in the analyses. Therefore, we interpret niche breadth differences between study sites as the result of variation in prairie-chicken diet sources. However, plant δ^13^C and δ^15^N signatures can be quite variable within and between landscapes, due to complex and poorly understood relations with soil and topographic variables [73], [74]. Hence our results may be also reflecting differences in habitat use, another facet of niche breadth. In addition, our goal was mainly to get comparative measures of trophic niche, rather than estimating the proportion of diet items; although the lack of a suitable baseline suggests a cautious interpretation of the results, we considered that baseline isotopic variance is unlikely to obscure the clear patterns identified here (see Text S1 for further discussion).

## Supporting Information

Figure S1 **Isotopic values (mean ± SE) for each of the 3 main diet categories and for male and female Greater Prairie-Chicken feathers in native prairie and agricultural mosaic.**
(TIF)

Table S1 **Stable isotopes of carbon and nitrogen (** ***δ*** **^13^C;** ***δ*** **^15^N; mean ± SD; N  =  sample size) in the main groups of vegetation sampled in the native prairie study site.** We also provide isotopic values of Greater Prairie- Chicken feathers (*δ* ^13^C; *δ* ^15^N; mean ± SD; N  =  sample size) in the same study site for comparison.
(DOCX)

Table S2 **Stable isotopes of carbon and nitrogen (** ***δ*** **^13^C;** ***δ*** **^15^N; mean ± SD; N  =  sample size) for sorghum in the native prairie and agricultural mosaic study sites.**
(DOCX)

Text S1 Isotopic baseline analysis.
(DOCX)
