# Correction: Effects of Sexual Dimorphism and Landscape Composition on the Trophic Behavior of Greater Prairie-Chicken

**DOI:** 10.1371/annotation/5a8bf4bc-b9bf-4feb-b887-e66cd14f85db

**Published:** 2014-01-02

**Authors:** Beatriz Blanco-Fontao, Brett K. Sandercock, José Ramón Obeso, Lance B. McNew, Mario Quevedo

A funding organization and grant were incorrectly omitted from the Funding Statement. The Funding Statement should read:

"The project was financially supported by a PFPU-MEC fellowship to BBF, grant CN-07-174, from the Asturian Environmental Agency to JRO, grant UNOV-08-MB-2 from Oviedo University to MQ and grant CGL2010-15990 (MICINN) to MJ Bañuelos. The funders had no role in study design, data collection and analysis, decision to publish, or preparation of the manuscript."

