# Supplementary figures and images for: Effects of Sexual Dimorphism and Landscape Composition on the Trophic Behavior of Greater Prairie-Chicken
Source: PLoS One. 2013 Nov 11;8(11):e79986. doi: 10.1371/journal.pone.0079986 (PMC3823567; doi:10.1371/journal.pone.0079986)

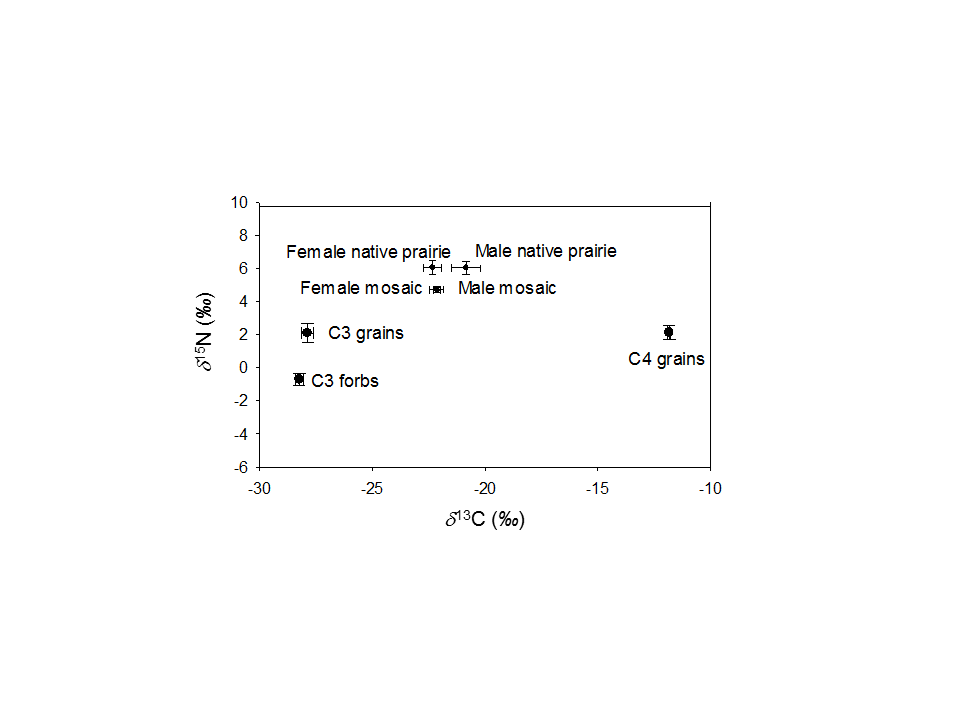

Supplement: Figure S1 — Isotopic values (mean ± SE) for each of the 3 main diet categories and for male and female Greater Prairie-Chicken feathers in native prairie and agricultural mosaic. (TIF) [file pone.0079986.s001.tif]
