# Supplementary material for: Effects of Sexual Dimorphism and Landscape Composition on the Trophic Behavior of Greater Prairie-Chicken
Source: PLoS One. 2013 Nov 11;8(11):e79986. doi: 10.1371/journal.pone.0079986 (PMC3823567; doi:10.1371/journal.pone.0079986)
Supplement: Table S1 — Stable isotopes of carbon and nitrogen ( δ 13C; δ 15N; mean ± SD; N = sample size) in the main groups of vegetation sampled in the native prairie study site. We also provide isotopic values of Greater Prairie- Chicken feathers (δ 13C; δ 15N; mean ± SD; N = sample size) in the same study site for comparison. (DOCX) [file pone.0079986.s002.docx]

Table S1

Stable isotopes of carbon and nitrogen (*δ*^13^C; *δ*^15^N; mean ± SD; N = sample size) in the main groups of vegetation sampled in the native prairie study site. We also provide isotopic values of Greater Prairie- Chicken feathers (*δ*^13^C; *δ*^15^N; mean ± SD; N = sample size) in the same study site for comparison.

| **Sample** | **N** | ***δ*^13^C** | ***δ*^15^N** |
| --- | --- | --- | --- |
| C3 grains | 6 | -27.88 ± 0.69 | 1.56 ± 0.72 |
| C4 grains | 12 | -11.82 ± 0.31 | 2.24 ± 1.85 |
| C3 forbs | 13 | -28.23 ± 0.67 | -0.69 ± 1.25 |
| Prairie -Chicken males | 22 | -21.00 ± 2.10 | 5.85 ± 1.17 |
| Prairie-Chicken females | 23 | -21.05 ± 2.04 | 5.78 ± 1.41 |
