# Supplementary material for: Effects of Sexual Dimorphism and Landscape Composition on the Trophic Behavior of Greater Prairie-Chicken
Source: PLoS One. 2013 Nov 11;8(11):e79986. doi: 10.1371/journal.pone.0079986 (PMC3823567; doi:10.1371/journal.pone.0079986)
Supplement: Table S2 — Stable isotopes of carbon and nitrogen ( δ 13C; δ 15N; mean ± SD; N = sample size) for sorghum in the native prairie and agricultural mosaic study sites. (DOCX) [file pone.0079986.s003.docx]

**Table S2**

Stable isotopes of carbon and nitrogen (*δ*^13^C; *δ*^15^N; mean ± SD; N = sample size) for sorghum in the native prairie and agricultural mosaic study sites.

| **Sorghum** | **N** | ***δ*^13^C** | ***δ*^15^N** |
| --- | --- | --- | --- |
| Native prairie | 6 | -11.74 ± 0.38 | 3.00 ± 1.27 |
| Agricultural mosaic | 4 | -11.92 ± 0.30 | 2.60 ± 0.84 |
