# Supplementary material for: Effects of Sexual Dimorphism and Landscape Composition on the Trophic Behavior of Greater Prairie-Chicken
Source: PLoS One. 2013 Nov 11;8(11):e79986. doi: 10.1371/journal.pone.0079986 (PMC3823567; doi:10.1371/journal.pone.0079986)
Supplement: Text S1 — Isotopic baseline analysis. (DOCX) [file pone.0079986.s004.docx]

**Text S1**

**Isotopic baseline analysis**

Vegetation collection and analysis

We collected vegetation samples randomly distributed in the native prairie study site during autumn 2008 coinciding with the feather synthesis for Greater Prairie-Chickens; these feathers were collected later in spring 2009 (see methods of the article). We grouped the sources of Prairie-Chickens diet in 3 categories reflecting most of the previously reported variation in their diets [1-5] and, showing a priori differences in the δ^13^C- δ^15^N isotopic values [6]: C3 grains consisting of corn *Zea mays* and sorghum *Sorghum* sp., C4 grains consisting of soy *Glycine max* and C3 forbs consisting of *Rosa* sp., *Solidago* sp, and *Rhus* sp.; see Table S1 for sample size. In addition, we collected C4 grains (sorghum) in the agricultural mosaic to provide comparison between study sites (normal distribution; t-test). Approx. 10 grains were collected for the grain categories and 5 cm of the tip of a branch/ plant for the C3 forbs category. Samples were dried at 60 ºC for 48 h, ground with a Retsch MM200 ball and stored in a paper envelope for isotopic analysis. The procedure followed for the isotopic analysis of vegetation samples was the same as for prairie-chicken tissues (see *Stable isotope analysis* section in the methods of the article for details).

*Baseline results*

Isotopic signatures for prairie-chicken diet sources are reported in Tables S1 and S2 and Figure S1.

We did not find differences between C4 grains in agricultural mosaic and native prairie (p> 0.1 in t-tests for δ^13^C and δ^15^N; see Table S2). Thus, we assumed similar isotopic signatures and variability for diet sources on both study sites.

*Endpoints for Greater Prairie-Chicken*

The representation of the consumer’s trophic niche is generally assumed to be dependent on the isotopic signatures of sources at the base of the food web, and therefore it should be interpreted on the basis of a baseline (see [7,8]). This isotopic baseline clearly facilitates the interpretation of results in food- web centered studies, especially when (1) the variability of primary producers is averaged up several trophic levels and (2) potential habitats or trophic resources clearly differ in the isotopic signatures (i.e. exclusively C3 or C4 plant systems, freshwater or marine food sources; [9]). However, this is not the case of our study system, where all the intrinsic variability of a fine scale mix of C3 and C4 crop and pasture grasses is integrated over a single trophic level [10].

Nonetheless, based on previous knowledge of Greater prairie-chicken diet [11], we collected and analyzed the major plant species looking for a proper baseline (see above). However, considering mean isotopic fractionation values reported in the literature (e.g., 3.4‰ for - δ^15^N and 0.4‰ for δ^13^C [12]), baseline cannot explain the physiological mechanism under our Greater-prairie chicken isotopic data. Fractionation is likely to be quite variable among groups and thus it may require specific empirical assessment for each trophic link of interest [13]. For example, isotopic distance between C3 grains and male Greater-Prairie-chicken feathers averages 6.88 units for δ^13^C and 4.22 for δ^15^N; values several units over the predictive power of the fractionation rates provided by the literature [12]. Moreover, herbivore diets may include a great variability of species of primary producers, (1) making difficult the sampling process and (2) remaining beyond the explanatory power of mixing models [14].

The focus of this article was in assessing the overall trophic niche for each sex, and intra-gender variability in resource use rather than detecting differences in the bulk diet composition of females and males. With this purpose we used Layman metrics for isotopic structure assessment of a population [15]. Layman measures rely on the variability of isotopic signatures; hence differences in the variability of source may be reflected in the consumer values and affect our results [7,15]. However, we assumed similar baselines between study sites on the basis of the lack of statistical differences between isotopic values of sorghum in the native prairie and agricultural mosaic (Table S2); we also added caution to the interpretation of the differences between the study sites.

Finally, in spite of all the drawbacks above mentioned, that supposedly would make difficult to find isotopic differences in population structure, we obtained markedly distinct isotopic niches between male and female tissues (except for blood) collected in native prairie and agricultural mosaic (Fig. 3). This could be biased by the confounding effect of landscape configuration. However, we minimized this bias by including landscape as an independent variable in the statistical analysis. Thus, we consider that baseline isotopic variance is unlikely to obscure the clear patterns identified in this article.

**Literature cited**

1. Mohler LL (1952) Fall and winter habits of Prairie-Chickens in southwest Nebraska. Journal of Wildlife Management 16: 9-23.

2. Yeatter RE (1943) The Greater Prairie-Chicken in Illinois. Illinois Natural History Survey Bulletin 22: 377-416.

3. Korschgen LJ (1962) Food habits of Greater Prairie-Chickens in Missouri. The American Midland Naturalist 68: 307-318.

4. Jones RE (1963) Identification and analysis of Lesser and Greater Prairie-Chicken habitat. Journal of Wildlife Management 25: 757-778.

5. Rumble MA, Newell JA, Toepfer JE (1988) Diets of Greater Prairie-Chickens on the Sheyenne National Grasslands. United States Department of Agriculture Forest Service General Technical Report RM-159: 49-54.

6. Carleton SA, Martínez del Rio C (2005) The effect of cold-induced increased metabolic rate on the rate of C-13 and N-15 incorporation in house sparrows (*Passer domesticus*). Oecologia 144: 226-232.

7. Matthews B, Mazumder A (2004) A critical evaluation of intrapopulation variation of delta C-13 and isotopic evidence of individual specialization. Oecologia 140: 361-371.

8. Newsome SD, del Rio CM, Bearhop S, Phillips DL (2007) A niche for isotopic ecology. Frontiers in Ecology and the Environment 5: 429-436.

9. Fry B (2008) Stable Isotope Ecology. Baton Rouge, LA: Springer Science.

10. Craine JM, Towne EG, Ocheltree TW, Nippert JB (2012) Community traitscape of foliar nitrogen isotopes reveals N availability patterns in a tallgrass prairie. Plant and Soil 356: 395-403.

11. Jonhson JA, Schroeder MA, Robb LA (2011) Greater prairie-chicken (*Tympanuchus cupido*). In: Poole A, editor. The Birds of North America Online. New York: Cornell Lab of Ornithology, Ithaca

12. Post DM (2002) Using stable isotopes to estimate trophic position: models, methods and assumptions. Ecology 83: 703-718.

13. Spence KO, Rosenheim JA (2005) Isotopic enrichment in herbivorous insects: a comparative field-based study of variation. Oecologia 146: 89-97.

14. Phillips DL, Gregg JW (2003) Source partitioning using stable isotopes: coping with too many sources. Oecologia 136: 261-269.

15. Layman CA, Arrington DA, Montana CG, Post DM (2007) Can stable isotope ratios provide for community-wide measures of trophic structure? Ecology 88: 42-48.
